# The BC ADPKD Network: A Comprehensive Provincial Approach to Support Specialized and Locally Delivered Multidisciplinary ADPKD Care

**DOI:** 10.1177/20543581211035218

**Published:** 2021-07-29

**Authors:** M. Bevilacqua, S. Gradin, J. Williams, A. Romann, C. Lo, O. Djurdjev, A. Levin

**Affiliations:** 1 Division of Nephrology, The University of British Columbia, Vancouver, Canada; 2 BC Renal, Vancouver, BC, Canada

**Keywords:** ADPKD (autosomal dominant polycystic kidney disease), standardization, clinical network, best practices, multidisciplinary clinic

## Abstract

**Purpose:**

With evolving evidence around the progression, assessment, and management of autosomal dominant polycystic kidney disease (ADPKD), care of the disease has become increasingly complex. Needs assessments in British Columbia (BC) described variability in knowledge and comfort with incorporating these new aspects of ADPKD care into clinical practice. Undercapture of early-stage ADPKD patients in existing renal databases was also identified as an unmet need.

**Sources of Information:**

A multidisciplinary group of clinicians and patient partners with interest and expertise in ADPKD and/or multidisciplinary kidney care informed the project work. An existing provincial renal database was used to support the provincial ADPKD registry.

**Methods:**

A formalized, comprehensive provincial ADPKD Network was created within the existing infrastructure of multidisciplinary kidney clinics (MDCs) in BC. The Network is coordinated provincially and implemented locally. It incorporates robust data collection, education, creation, and dissemination of dedicated clinical tools; collaboration between clinics and clinicians across the province; and ongoing evaluation and continuous quality improvement.

**Key Findings:**

Over the 5 years since its inception, the BC ADPKD Network has enabled increased and earlier identification of British Columbians living with ADPKD and a shift in practice toward increased and earlier enrollment of ADPKD patients into MDCs. A host of tailored ADPKD clinical tools have been created and implemented in all MDCs across the province to support existing MDC staff in the delivery of more standardized and specialized ADPKD care. A collaborative provincial clinician network founded on Local Clinical Champions has been established to support ongoing experience sharing between clinics. An evaluation framework has been established to evaluate outcomes and enable ongoing refinement of the Network.

**Limitations:**

The provincial ADPKD registry is undergoing enhancements to enable more comprehensive capture of APDKD-specific information such as total kidney volume and genetic results, but at present, this remains a limitation. It remains to be seen whether the activities of the ADPKD Network will improve long-term clinical outcomes and care experiences of patients living with ADPKD, and a long-term sustainability assessment of this model of care will be required.

**Implications:**

The structure, tools, and coordinated and collaborative clinician network established through this comprehensive provincial ADPKD Network may be valuable in addressing the variability and gaps in existing ADPKD care while allowing patients and families across BC to receive enhanced care locally, in their usual kidney care environments.

## Abrégé

**Justification:**

L’évolution des données entourant la progression, l’évaluation et la prise en charge de la polykystose autosomique dominante (ADPKD) complexifie de plus en plus son traitement. En Colombie-Britannique (C.-B.), une évaluation des besoins a décrit la variabilité des connaissances et le niveau de confort quant à l’intégration de ces nouveaux aspects des soins pour l’ADPKD dans la pratique clinique. La capture des patients atteints d’ADPKD à un stade précoce dans les bases de données rénales existantes a également été reconnue comme un besoin non satisfait.

**Sources:**

Les travaux ont été orientés par un groupe multidisciplinaire de cliniciens et de patients partenaires possédant une expertise dans le domaine de l’ADPKD ou dans les soins rénaux multidisciplinaires. Une base de données provinciale existante sur les maladies rénales a été utilisée en appui au registre provincial de l’ADPKD.

**Méthodologie:**

Un réseau provincial complet et structuré pour l’ADPKD a été créé au sein de l’infrastructure existante des cliniques rénales multidisciplinaires (CRM) de la Colombie-Britannique. Le réseau est coordonné à l’échelle provinciale et mis en œuvre localement. Le réseau englobe une collecte rigoureuse des données, de l’éducation, la création et la diffusion d’outils cliniques dédiés, la collaboration entre les cliniques et les cliniciens de toute la province, de même que l’évaluation et l’amélioration continues de la qualité.

**Principaux résultats:**

Au cours des cinq années qui ont suivi sa création, le réseau ADPKD de la C.-B. a permis d’identifier en plus grand nombre et plus précocement les Britanno-Colombiens vivant avec l’ADPKD. On a également pu observer un virage dans la pratique vers une plus vaste et une plus précoce inclusion des patients atteints d’ADPKD dans les CRM. De plus, une foule d’outils cliniques sur mesure ont été créés et mis en œuvre dans tous les CRM de la province afin d’appuyer le personnel des CRM existantes dans la prestation de soins normalisés et plus spécialisés pour l’ADPKD. Un réseau coopératif provincial de cliniciens, fondé sur les champions cliniques locaux, a été créé pour favoriser le partage continu de l’expérience entre les cliniques. Enfin, un cadre d’évaluation a été établi pour examiner les résultats et permettre l’amélioration continue du réseau.

**Limites:**

Le registre provincial de l’ADPKD fait présentement l’objet d’améliorations qui permettront une saisie plus complète de l’information spécifique à l’APDKD, notamment le volume rénal total et les résultats génétiques, mais à l’heure actuelle, ceci demeure une limite. Reste à voir si les activités du réseau ADPKD permettront d’améliorer les résultats cliniques à long terme et les expériences de soins des patients vivant avec l’ADPKD. Une évaluation à long terme de la durabilité de ce modèle de soins sera nécessaire.

**Conclusion:**

La structure, les outils et le réseau coordonné et collaboratif de cliniciens mis en place par le biais de ce réseau provincial sur l’APDKD peuvent être utiles pour rendre compte de la variabilité et combler les lacunes des soins existantes, tout en permettant aux patients et aux familles de la Colombie-Britannique de recevoir localement de meilleurs soins, soit dans leur milieu de soins rénaux habituel.

## What Was Known Before

With evolving evidence around disease progression, assessment, and management, providing specialized autosomal dominant polycystic kidney disease (ADPKD) care has become increasingly complex in recent years. Several provider surveys and needs assessments have shown variability in clinical resource availability as well as provider knowledge and comfort with incorporating new aspects of ADPKD care into routine clinical practice.

## What This Adds

A detailed description of a provincially coordinated and locally delivered approach to address variability and gaps in ADPKD care by enabling care providers in diverse practice settings to provide standardized, best practice ADPKD care within existing renal care settings. The creation, administrative structure, components, and activities of the BC ADPKD Network are described in sufficient detail to support replication of a similar coordinated network of care in other jurisdictions.

## Background

Autosomal dominant polycystic kidney disease (ADPKD) is the most common inherited kidney disorder and the fourth leading cause of kidney failure in Canada and internationally.^1,2^ In recent years, understanding of the progression, prognostication, and management of ADPKD has greatly advanced.^3^ Nonetheless, translation of this knowledge and access to specialized ADPKD care vary greatly; in Canada, there is variation between provinces, institutions, and providers.^4,5^ The lack of consistent resources and guidelines for treating ADPKD has been identified as a priority among Canadian nephrologists,^4^ and with differences in practice size and setting as well as exposure to ADPKD patients, nephrologists’ ability to stay up to date with such guidelines and implement new ADPKD management strategies into practice is variable.^4,5^

BC Renal (BCR) is the organization responsible for coordinating kidney care across British Columbia (BC), a large and geographically diverse Canadian province. Recognizing these challenges, BCR launched a provincial strategy to enhance ADPKD management in 2015 with the goal of enabling access to best available ADPKD care wherever patients live and receive their kidney care. It is the purpose of this article to describe the development and implementation of this first in kind comprehensive provincial Network, its creation, administrative structure, key components, and results to date.

### Needs Assessments and Requirements of a Provincial ADPKD Network

Between 2015 and 2018, targeted needs assessments and practice surveys in BC revealed substantial variability in ADPKD management across the province in terms of location of care, services offered, provider familiarity with modern ADPKD assessment and management strategies, and the application of recommended treatments.^5^

In addition, it was identified that data were lacking regarding ADPKD and its management in BC. Using BCR’s existing database of patients, in 2015 it was noted that there were 308 dialysis and transplant patients, but only 237 ADPKD patients were at earlier stages, a number that was a clear underestimate of the number of British Columbians living with ADPKD in terms of both their prevalence in renal replacement treatment programs and based on population estimates.^1^ These findings were further validated through dedicated focus group sessions with BC nephrologists and allied health care professionals, where it was determined that many nephrologists had not seen the need to register these patients in the provincial database, nor expose them to multidisciplinary clinic (MDC) resources.

**Figure 1.**
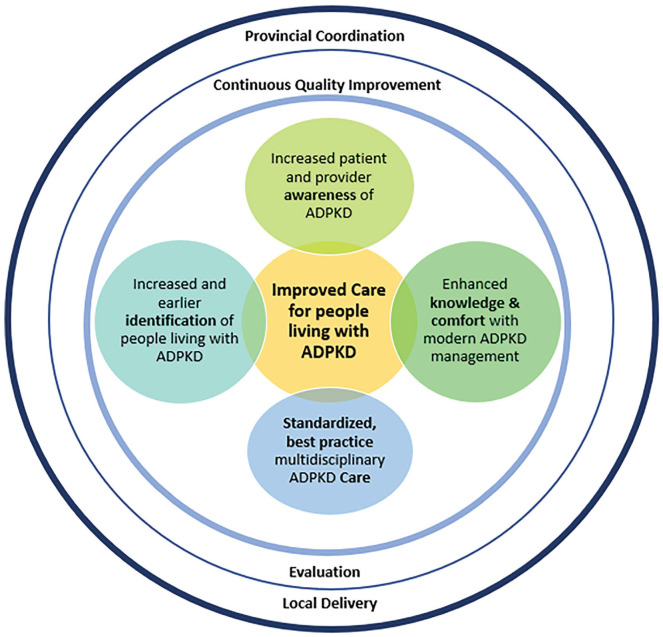
Components and objectives of the ADPKD Network. *Note.* ADPKD = autosomal dominant polycystic kidney disease.

Based on these identified needs, the following key components of a provincial ADPKD Network were identified (Figure 1):

1. A formalized provincial structure for project planning and oversight;
2. Development of a comprehensive provincial ADPKD patient registry;
3. A synthesis of best-practice guidance and supporting clinical tools into a practical format for delivery of ADPKD care, leveraging existing care infrastructures;
4. A formalized network of interested care providers to promote ongoing experience sharing between clinics and providers;
5. Educational tools and resources for physicians, clinic staff, and patients and families; and
6. An evaluation framework to inform refinement and continuous quality improvement of ADPKD care delivery.

Objectives and example activities for each of these components are shown in Table 1.

**Table 1.** Key Components, Objectives and Activities of the Provincial ADPKD Network.

Network component	Objectives	Examples
Provincial project planning and oversight	Establish an ADPKD advisory group with representation from interdisciplinary team members from MDCs across the province, patient partners, and other identified stakeholders to support development and implementation of the ADPKD Network activities	● Quarterly meetings to discuss ADPKD initiative progress and gain feedback on approach to specific activities ● Smaller working groups established for focused initiatives within the broader ADPKD initiative, eg, ADPKD treatments, ADPKD Imaging protocols, Standardized ADPKD labs, Evaluation, Patient resources
Provincial ADPKD patient registry	Establish a comprehensive provincial registry of all British Columbians living with ADPKD to inform practices, support quality improvement and research efforts.	● Leverage existing BCR provincial-wide database to capture identification of patients living with ADPKD ● Engage private nephrologist offices to register earlier stage ADPKD patients
Best-practice guidelines and accompanying clinical tools for standardized ADPKD care	Develop and implement a best practice guideline based on best available evidence and in alignment with Kidney Care Clinic best practices.	● Discipline-specific working groups from across the province as well as a larger working group to compile the full set of guidelines ● Resources published on the BCR website, communicated to clinics ● ADPKD local clinical champions identified in each Health Authority Renal Program (HARP) to support local implementation efforts, target implementation in all 14 kidney clinics
Educational resources	Develop and disseminate education tools and resources to build capacity of nephrologists, nurses, dietitians, social workers, and pharmacists to identify and deliver ADPKD care in collaboration with other health care disciplines.	● Targeted education to BC MDC staff, nephrologists in a variety of formats ● Targeted education and collaboration with external providers where applicable (eg, radiology, genetics, primary care)
Clinical experience sharing	Foster a network of interested ADPKD clinicians across the province to share experiences and collaborate on best practice and emerging aspects of ADPKD care	● Ongoing quarterly local clinical champion webinars ● Periodic sessions for larger nephrology community (eg, province wide rounds)
Evaluation of the provincial ADPKD strategy	Share experience from BC ADPKD initiatives locally and disseminate broadly; inform ongoing quality improvement of ADPKD care	● An evaluation strategy grounded in a project logic model to measure initiative’s overall success ● Inclusion of ADPKD in existing BCR metrics (eg, existing MDC indicator report)
Knowledge generation and translation	Contribute to enhanced BCR knowledge sharing and research efforts.	● Increase involvement of ADPKD patients across BC in research efforts (internal and multisite) ● Share results through publications/meetings/conferences in and outside of BC and publications

*Note.* ADPKD = autosomal dominant polycystic kidney disease; BCR = BC Renal; MDC = multidisciplinary clinic.

## Content and Construction of the ADPKD Network

### Project Planning and Oversight

A provincial ADPKD Advisory Group consisting of nephrologists, allied health professionals across health authorities and disciplines, and multiple patient partners was created and tasked with oversight of the Network. This advisory group was formed under the existing structure of the provincial Kidney Care Committee, which oversees delivery of MDC care in BC (Supplemental Figure 1). A guiding principle for this group was to establish tools, processes, and workflows that were standardized yet adaptable to diverse clinical practice settings across the province. This advisory group supported the creation of dedicated working groups to complete programs of work related to key deliverables as outlined above. A dedicated project team, including a medical lead, project manager, and administrative and analytics support, was established.

### Creation of the ADPKD Registry Within an Existing Provincial Database

BC has an existing provincial database for kidney patients known as the Patient Records, Outcome and Management Information System (PROMIS). Patients are registered in PROMIS once they access BCR-funded medications or clinical services such as MDCs or kidney replacement treatment. Although there is no threshold level of kidney function to access MDC clinics in BC, many nephrologists had historically not considered the value of MDC care for earlier stages of ADPKD. With improved understanding of the value of earlier assessment and follow-up,^3,4^ and to determine the true burden of disease for planning and forecasting purposes, the Advisory Group agreed that a comprehensive ADPKD registry should be developed within PROMIS, including ADPKD patients not yet enrolled in MDCs. As this registration process represented a change in workflow, a targeted effort was made to identify all private nephrology offices in the province, develop a streamlined registration process, and provide training to staff in those settings. A small time-limited financial incentive was offered for this process development and change management with the understanding that thereafter this would become a routine operational work item for staff in these offices. The only requirement for inclusion in the ADPKD registry is registration in PROMIS with a primary diagnosis of ADPKD; this diagnosis is provided by the patient’s treating nephrologist. Once a patient is registered, even if they transition between different programs or clinical locations, as long as they remain in BC, they remain in the ADPKD registry.

### Development and Dissemination of BC Best Practices for Care of ADPKD and Associated Clinical Tools

Using a provincial template previously used to describe “Best Practices in CKD,”^6^ the working groups developed a similar document but uniquely focussed on the management of ADPKD, within the context and resources of existing MDCs. This was not a formal guideline development process, but rather a process of collating existing literature and guidelines and outlining their integration into existing care models within BC. The working group consisted of all professional disciplines already represented in the MDCs with each discipline focusing on components relevant to their scope of practice. The disciplines represented in this way were physicians, pharmacists, registered nurses, dietitians, social workers, administrative, and clerical staff.

The development of the guidance documents was predicated on the need to complement rather than replace existing documents for more “generic” CKD care,^6^ emphasizing that with proper support ADPKD-specific care can be delivered within existing MDC settings. Integration of processes within existing care infrastructures, workflows, and funding models was prioritized; there was a conscious effort to avoid the creation of standalone clinics outside of the existing MDC network, which may have led to fractionation of care teams and flow. The resulting guidance document focuses on unique aspects of care specific to ADPKD patients while highlighting similarities of care to the general CKD population the MDCs are familiar with. Clinical tools to support ADPKD-specific care items were created. Where care tasks may be beyond the scope of MDC staff, links and collaboration with external providers is encouraged. For example, as some components of genetic testing, counseling, and reproductive options are generally outside the comfort of many MDC staff, links have been made with medical genetics providers in BC to promote access to these services for ADPKD patients and families across the province, and resources are under development to standardize this process.

The ADPKD best practices document is based on current available literature. Where no literature exists, the working groups assessed existing practices across the network and developed consensus guidance that would be feasible and practical for MDC staff. As an example, the literature does not provide clear guidance as to intracranial aneurysm monitoring.^7^ To address this uncertainty, the group focussed on reducing variability and testing at consistent intervals so as to enable MDC staff’s ability to ensure testing and follow-up, which has been shown to be an effective strategy to ensure follow-up and testing in other areas.^8^ For all recommendations, regular literature review and planned updates of these best practice recommendations have been embedded in the process, and all documents are dated to this effect.

To increase awareness of new guidelines and improve knowledge transfer to frontline staff, the ADPKD strategy includes the development of an educational session series targeted to existing CKD staff, delivered by provincial leaders. These sessions were targeted to nurses, pharmacists, and dietitians cognizant of specific scopes of practice and are available in different formats, including large group rounds, webinars, small group discussions, and in-person sessions.

ADPKD “Local Clinical Champions” were recruited from the pool of clinicians from each of the 14 MDCs across BC to assist with on the ground implementation of best practices and new clinic tools. These local champions serve as key conduits assisting with dissemination and implementation of new information and tools. They are also the individuals who serve to link clinics across the province, fostering regular collaboration and experience sharing. They are integral components of both continuous quality improvement and long-term sustainability of the ADPKD Network.

While the Network is predicated on local care delivery, in some situations providers choose to refer complex or challenging cases to nephrologists and centres with more experience. When this occurs, the strategy used is one of co-management wherein the more experienced providers provide this consultation, but the day-to-day and longitudinal care occurs at the patient’s local MDC.

### Development of a Dedicated Evaluation Framework

**Figure 2.**
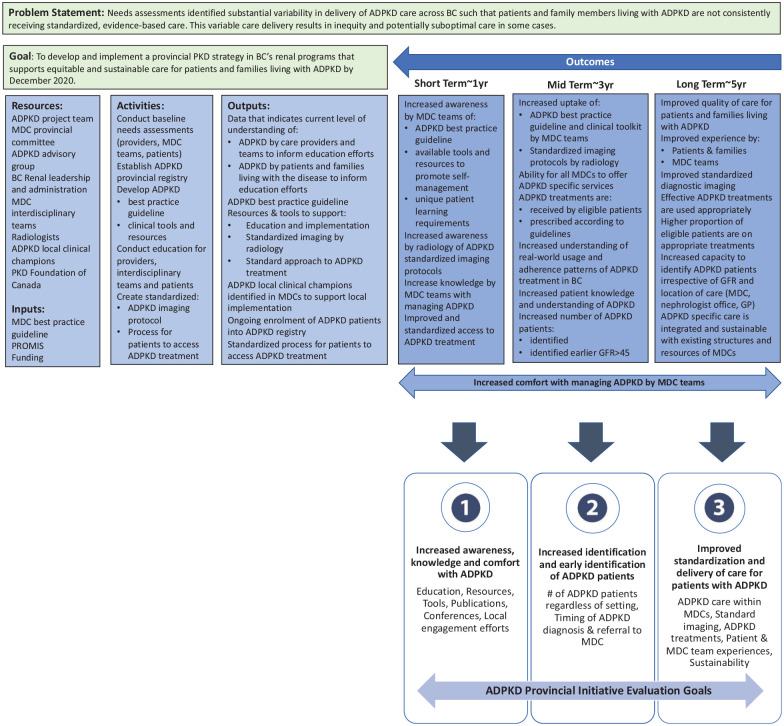
ADPKD Network evaluation logic model and outcomes. *Note.* ADPKD = autosomal dominant polycystic kidney disease; BC = British Columbia; PKA = polycystic kidney disease; PROMIS = Patient Records, Outcome and Management Information System; MDC = multidisciplinary kidney clinics; GP = general practitioner; GFR = glomerular filtration rate.

A multimodal evaluation framework was developed via a dedicated evaluation working group. The plan was based on a project logic model developed to inform clearly defined evaluation goals and objectives for this initiative (Figure 2). We created realistic and achievable short term, mid-term and long-term outcomes including a focus on sustainability. Three key ADPKD initiative evaluation goals were defined to inform the development of the evaluation framework and to ensure alignment: 1) promoting staff awareness, knowledge and comfort in managing ADPKD, 2) enabling comprehensive identification of ADPKD patients in BC and 3) standardization of ADPKD care delivery. The goals created a foundation in which to define specific evaluation activities and objectives. In addition to these goals, in order to ensure sustainability of this initiative, the NHS sustainability model^9^ will be utilized.

## Current State of the ADPKD Network and Findings to Date

### Increased and Earlier Registration of ADPKD Patients in BC

Between 2015 and 2020, the number of ADPKD patients registered in PROMIS increased from 547 to 1076; this includes patients registered either in a multidisciplinary renal clinic or in a private nephrologist’s office. From 2015 to 2020, a greater proportion of this registration occurred in individual nephrologists’ offices such that this now comprises over 60% of total patient registrations; this trend continued well after the time-limited funding for registration was discontinued in 2018.

**Figure 3.**
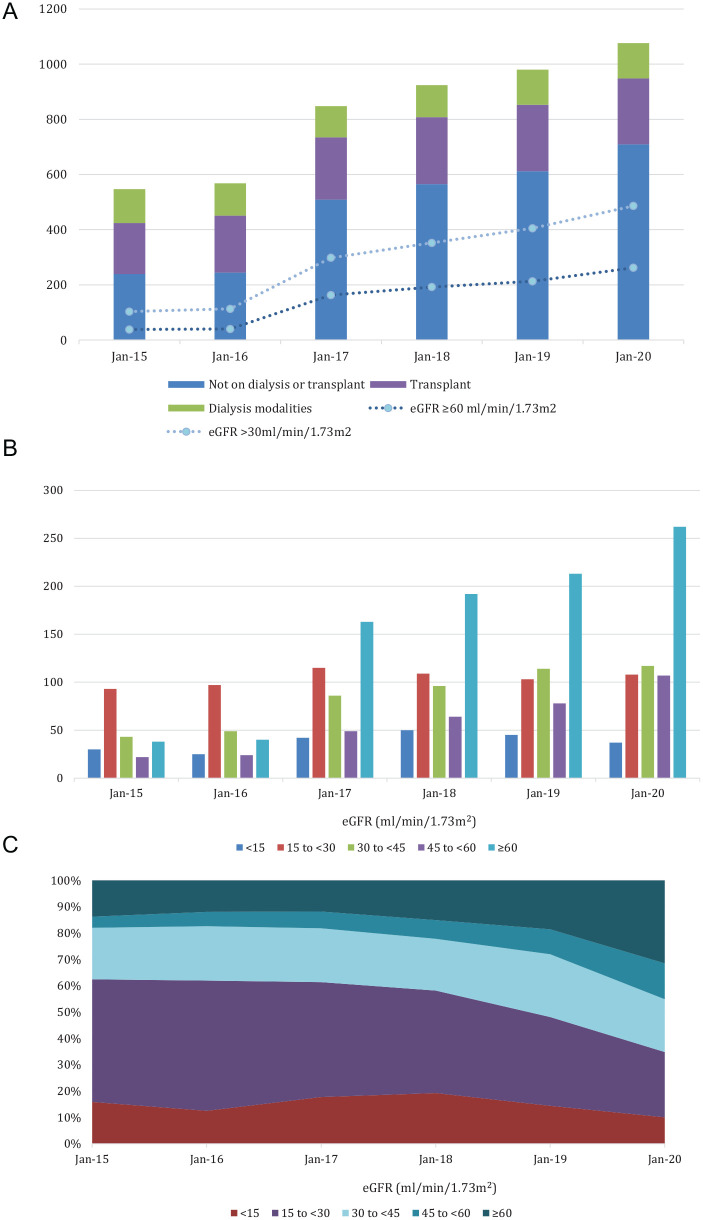
ADPKD patients registered 2015-2020. All eGFR values reported are the values for prevalent patients at the start of each period. (A) ADPKD patients registered by treatment program and number of registered patients with eGFR >30 and >60 mL/min/1.73 m^2^. (B) Level of kidney function of ADPKD patients registered prior to dialysis or transplant. (C) Level of kidney function of ADPKD patients followed in multidisciplinary kidney clinics. *Note.* ADPKD = autosomal dominant polycystic kidney disease; eGFR = estimated glomerular filtration rate.

Since the initiative was launched, the largest increase in patient registration has been those in earlier stages; the numbers of nondialysis or transplant patients increased from 239 to 709 for a 197% increase and the subset of those with eGFR >30 mL/min/1.73 m^2^ increased from 103 to 486 representing a 372% increase, whereas those with eGFR ≥ 60 mL/min/1.73 m^2^ increased from 38 to 262 representing a 589% increase (Figure 3A and B). As a comparison, over the same period, the overall number of nondialysis or transplant patients registered in PROMIS increased 13 136 to 16 111 for a 23% increase and the subset with eGFR >30 mL/min/1.73 m^2^ increased from 6319 to 7518 for a 19% increase, whereas those with eGFR ≥60 mL/min/1.73 m^2^ increased from 918 to 1513 representing a 65% increase.

Numbers of these earlier-stage ADPKD patients who were enrolled and followed in MDCs also increased. In 2015, there were 71 patients with eGFR > 30 mL/min/1.73 m^2^ and 26 patients with eGFR ≥ 60 mL/min/1.73 m^2^ followed in MDCs, respectively, representing 36% and 13% of the ADPKD patients followed in kidney clinics; in 2020, there were 234 patients with eGFR > 30 mL/min/1.73 m^2^ and 113 patients with eGFR ≥60 mL/min/1.73 m^2^, respectively, representing 63% and 31% of all ADPKD patients followed in MDCs (Figure 3C). As a comparison, for the general MDC clinic population in 2020, the proportion with eGFR >30 mL/min/1.73 m^2^ is 47% and the proportion with eGFR >60 mL/min/1.73 m^2^ is 5%, figures that are essentially unchanged over the same 5-year period. From 2015 to 2020, the median age at registration for ADPKD patients also decreased from 60 to 54 while over that same period the median age at registration for the general CKD cohort remained constant at 70 to 71 years.

It should be noted that in BC only nephrologists may refer to MDCs; general practitioners are unable to access MDCs directly. Registration in an MDC is done at the nephrologist’s discretion based on their opinion as to whether the patient would benefit from multidisciplinary services; there is no eGFR threshold or other strict criteria for MDC enrollment. This is not unique to ADPKD as the same is true for MDC enrollment of any CKD patient in BC, regardless of underlying diagnosis.

#### Content and implementation of ADPKD best practice guidelines and associated clinical tools

The best practices document includes recommendations tailored to the ADPKD population cared for within MDCs; Table 2 outlines the contents of this document. A host of complementary clinical tools and forms were developed to support knowledge dissemination, reduce variability of care delivery, or both (Table 2). The best practice document and all the associated clinical tools are freely available on the website www.bcrenal.ca. Where possible, patient-facing materials were developed to complement the staff tools. All new tools were created in direct collaboration with patient partners.

**Table 2.** Content of ADPKD Best Practice Guidelines and Associated New Clinical Tools Developed (All Documents Freely Available on www.bcrenal.ca).

Best practice guideline section	Associated clinical tools
Referral to MDC	None
Tasks and timelines for patients with ADPKD	None; similar to BC Renal Best Practices for Kidney Care Clinics
Patient education, assessment, active monitoring, goal setting and psychosocial support	Kidney Care Clinic: Learning Needs Questionnaire for New Patients with ADPKD Pain and Autosomal Dominant Polycystic Kidney Disease Pain Diary for Autosomal Dominant Polycystic Kidney Disease
Screening and testing	Screening and Testing for Autosomal Dominant Polycystic Kidney Disease Pregnancy and Family Planning in Autosomal Dominant Polycystic Kidney Disease In process: Standardized Application and Criteria for Genetic Testing in ADPKD
Renal imaging	Approach to Renal Imaging in ADPKD Standardized Measurement instructions for TKV in ADPKD UBC Ultra-Low-dose CT protocol for TKV assessment in ADPKD (in process)
Dietary support	Staff Guide: Dietary Recommendations for Patients with Autosomal Dominant Polycystic Kidney Disease (ADPKD) Diet Changes for Adults with Polycystic Kidney Disease (for patients) Diet Changes for Adults with Polycystic Kidney Disease Taking Tolvaptan (for patients)
Management of patients treated with tolvaptan	Application for tolvaptan in ADPKD Tolvaptan Frequently Asked Questions (for patients) Tolvaptan Frequently Asked Questions (for prescribers) Tolvaptan Pharmacy Information Sheet Standardized lab requisition for ADPKD patients on tolvaptan
Management of blood pressure	Supporting evidence: Blood pressure monitoring and targets in ADPKD Supporting evidence: Antihypertensive agents in ADPKD patients
Management of lipids	Supporting evidence: lipid lowering therapy in ADPKD patients
Clinic resources, documentation for patients	Information at a Glance: Patients with ADPKD (Addendum to KCC Kardex) Kidney Care Clinic: Clinic Visit Form for Patients with ADPKD

*Note.* TKV = total kidney volume; MDC = multidisciplinary kidney clinics; CT = computed tomography.

It is worth mentioning that while these guidelines and tools are standardized and developed centrally, flexibility was given, and adaptations or modifications of the new guidelines and tools were encouraged at the local level in a way that best complements the needs and resources of local kidney clinics and patients. This is no different from general MDC care in BC where local variation is supported and encouraged. As examples of localized variation in implementation of ADPKD-specific resources, some have cohorted patients to specific days while others have them interspersed with regular CKD patients, and some have dedicated multidisciplinary staff while others have a more flexible team that provides both ADPKD and general CKD care; these and other variations in implementation details are encouraged to enable each MDC to provide ADPKD care in a way that is feasible and sustainable in their individual environment.

## Discussion

We describe here the objectives, development, structure, and implementation of a first in kind comprehensive provincial approach to ensuring consistent, equitable, and sustainable best-practice care for patient and families living with ADPKD. In the short term through the ADPKD Network, we have demonstrated an ability to identify larger numbers of ADPKD patients with a particular focus on those at earlier disease stages and have shifted practice such that more of these patients early in their disease trajectory are now being followed in MDCs.

This change in care delivery and practice patterns over the 5 years since the Network has been active will require further evaluation as to whether patient or system outcomes have been impacted. To this end, in the longer term, through the Network’s established evaluation framework, we will report on demographics, clinical parameters, treatment and medication usage, outcomes and other key indicators to provide real-time feedback to clinicians and patients, as well as contributing to better understanding of the disease itself. The use of these data will inform continuous quality improvements in ADPKD care, serve to support ongoing research efforts, and facilitate patient identification for future trials.

The approach to implementing best practice in ADPKD care described herein is different than many other descriptions that tend to focus on the creation of specialized centers of excellence for ADPKD care which function as standalone entities, often in urban or academic centers.^7,10^ While such centers certainly have an important role and there are self-identified individuals with greater ADPKD experience and knowledge in BC, rather than having all ADPKD patients see these few providers in specific locations, the model pursued in this Network is that the more knowledgeable providers are engaged and serve as reference points for the other nephrologists and MDCs across the province who in turn deliver care to ADPKD patients in their own clinics. BCR has had similar success in the past with a similar approach to care delivery in the creation of a provincial glomerulonephritis network,^11^ which served as a model for the ADPKD Network. This promotes accessibility across large geographical areas and is felt to be a more equitable approach. In addition, providing these services within the existing MDCs in an integrated manner provides a smoother transition of care as these patients progress and ultimately move on to other clinics and treatment modalities. Finally, providing specialized ADPKD care in an integrated manner provides a model for these kidney clinics wherein specialized disease-specific care is layered on top of more general care aspects to create more patient-centered care offerings in MDCs.

There are many factors that facilitated the development of this ADPKD network. The first is that BC MDCs do not require a specific level of kidney function before registration; indeed, the existing *BC Best Practices for Kidney Clinics*^6^ emphasizes that some patients, specifically including those with inherited kidney diseases, may benefit from earlier registration in kidney clinics.^6^ Second, BCR has an existing infrastructure to support MDCs as well as an overarching provincial structure with a mandate of supporting consistent and equitable care across the province. A third enabling factor in BC is the activity-based funding model used for all renal care in the province; this model has been described in detail elsewhere,^12^ but in essence allows funding to follow patients proportionate to the number of patient years accrued in the clinic, thus ensuring proportionate funding for any newly identified and enrolled group of patients. Through this model, patients are funded at a blended rate commensurate to their level of kidney function that reflects the expected level of required services. From its inception and validation, this model has specifically included patients who may require earlier access to MDC care such as those with glomerulonephritides and inherited renal diseases,^12^ so no new funding models or financial evaluations were required to support earlier MDC registration of ADPKD patients. Taken together, these enabling factors supported development of the Network and tailored clinical tools that could be implemented in diverse practice settings and delivered within existing clinic structures across the province.

## Limitations

There remain limitations to our approach. Given differences in patient load, number of health care professionals, and other workload factors, there has been some adaptation of clinical processes and thus variation; we have not yet determined whether or how that variation influences outcomes. Furthermore, at present, the registry requires further enhancements to comprehensively capture key ADPKD-specific information such as TKV and genetic results. In the first instance, we are able to report only on patterns of patient registration and MDC enrollment, but the results described herein are early stages, and the impact of this initiative on long-term clinical outcomes and patient care experiences remains unknown. An objective of the ADPKD Network is to continue to strive to answer these and other questions. The ADPKD network aims to be iterative, building on emerging evidence in ADPKD care, and assessing what is working well, what is not, and where refinements are needed. As these refinements continue and these care networks continue to evolve, a long-term sustainability assessment of this model of care will also be required.

## Conclusion

The BC ADPKD Network described herein represents creation and implementation of a first in Canada comprehensive provincial approach to enhancing care of patients living with ADPKD. The Network is predicated on leveraging and empowering existing local care providers and clinics to provide best practice ADPKD care through support from a collaborative network of dedicated clinicians and development and implementation of tailored clinical tools. A core component of this work is a provincial registry that has enabled earlier and increased identification of ADPKD patients for planning and management purposes. The provincial ADPKD registry is also a foundational component in the ongoing evaluation of practice patterns and outcomes to enable further refinement of ADPKD services. Taken together, the structure of the Network may be valuable in addressing the variability and gaps in existing ADPKD care while allowing patients and families across BC to receive enhanced care in their usual kidney care environments.

## Supplemental Material

sj-pdf-1-cjk-10.1177_20543581211035218 – Supplemental material for The BC ADPKD Network: A Comprehensive Provincial Approach to Support Specialized and Locally Delivered Multidisciplinary ADPKD Care Click here for additional data file. Supplemental material, sj-pdf-1-cjk-10.1177_20543581211035218 for The BC ADPKD Network: A Comprehensive Provincial Approach to Support Specialized and Locally Delivered Multidisciplinary ADPKD Care by M. Bevilacqua, S. Gradin, J. Williams, A. Romann, C. Lo, O. Djurdjev and A. Levin in Canadian Journal of Kidney Health and Disease
