# Supplementary material for: The BC ADPKD Network: A Comprehensive Provincial Approach to Support Specialized and Locally Delivered Multidisciplinary ADPKD Care
Source: Can J Kidney Health Dis. 2021 Jul 29;8:20543581211035218. doi: 10.1177/20543581211035218 (PMC8330454; doi:10.1177/20543581211035218)

Supplement

Supplemental Figure 1: Governance and structure of the APDKD Network within existing BC Renal infrastructure

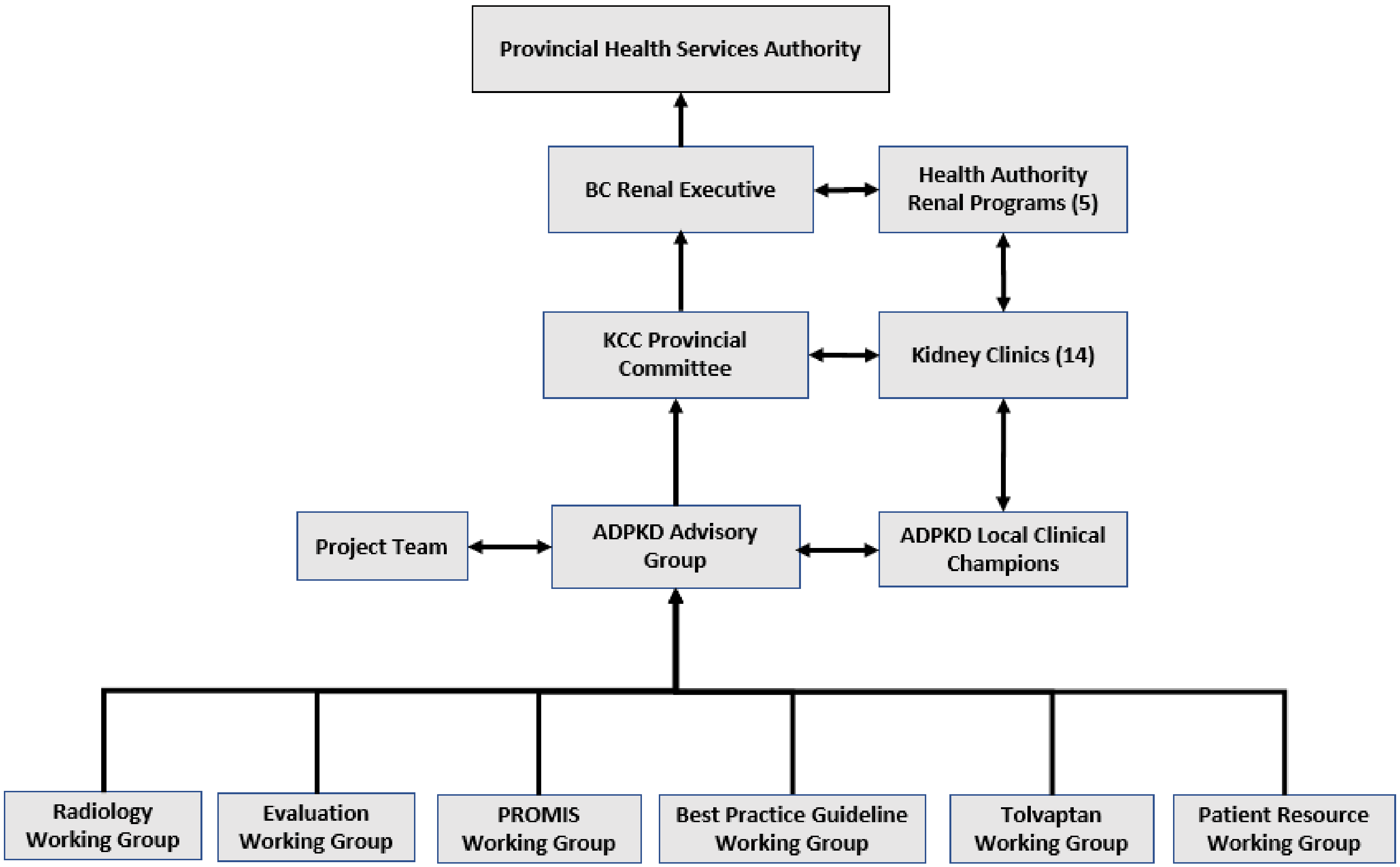

Supplement: sj-pdf-1-cjk-10.1177_20543581211035218 – Supplemental material for The BC ADPKD Network: A Comprehensive Provincial Approach to Support Specialized and Locally Delivered Multidisciplinary ADPKD Care [file sj-pdf-1-cjk-10.1177_20543581211035218.pdf]
